# Experimental Study of Membrane Fouling during Crossflow Microfiltration of Yeast and Bacteria Suspensions: Towards an Analysis at the Microscopic Level

**DOI:** 10.3390/membranes3020044

**Published:** 2013-05-10

**Authors:** Ines Ben Hassan, Monia Ennouri, Christine Lafforgue, Philippe Schmitz, Abdelmoneim Ayadi

**Affiliations:** 1 LRAE (Laboratoire Radio analyses et Environnement), Ecole nationale d‘ingénieurs de Sfax., Sfax BP3038, Tunisie; E-Mails: inesbenhassan@yahoo.fr (I.B.H.); moneim.ayadi@enis.rnu.tn (A.A.); 2 Université de Toulouse, INSA, UPS, INP, LISBP, 135 Avenue de Rangueil, Toulouse F-31077, France; E-Mail: philippe.schmitz@insa-toulouse.fr; 3 INRA (Institut National de la Recherche Agronomique), UMR792, Ingénierie des Systèmes Biologiques et des Procédés, Toulouse F-31400, France; 4 CNRS (Centre National de la Recherche Scientifique), UMR5504, Toulouse F-31400, France; 5 LAA (Laboratoire Analyses Alimentaires), Ecole nationale d‘ingénieurs de Sfax., Sfax BP3038, Tunisie; E-Mail: ennouri_monia@yahoo.fr; 6 Institut supérieur des Sciences Appliquées et de Technologie de Mahdia, Hiboun Sidi Massoud Mahdia 5111, Tunisie

**Keywords:** microfiltration, *Saccharomyces cerevisiae*, *Escherichia coli*, filtration resistances, filtration chamber design, direct observation

## Abstract

Microfiltration of model cell suspensions combining macroscopic and microscopic approaches was studied in order to better understand microbial membrane fouling mechanisms. The respective impact of *Saccharomyces cerevisiae* yeast and *Escherichia coli* bacteria on crossflow microfiltration performances was investigated using a multichannel ceramic 0.2 µm membrane. Pure yeast suspensions (5 µm ovoid cells) and mixtures of yeast and bacteria (1 to 2.5 µm rod shape cells) were considered in order to analyse the effect of interaction between these two microorganisms on fouling reversibility. The resistances varied significantly with the concentration and characteristics of the microorganisms. Membrane fouling with pure yeast suspension was mainly reversible. For yeast and bacteria mixed suspensions (6 g L^−1^ yeast concentration) the increase in bacteria from 0.15 to 0.30 g L^−1^ increased the percentage of normalized reversible resistance. At 10 g L^−1^ yeast concentration, the addition of bacteria tends to increase the percentage of normalized irreversible resistance. For the objective of performing local analysis of fouling, an original filtration chamber allowing direct *in situ* observation of the cake by confocal laser scanning microscopy (CLSM) was designed, developed and validated. This device will be used in future studies to characterize cake structure at the microscopic scale.

## 1. Introduction

Cell filtration technology is widely used in biotechnology, food processing and the beverage industry, in drinking water production as well as for wastewater treatment [1,2,3] or as a preliminary step in purification processes [4,5]. The main factor limiting the membrane performances in these applications is their propensity to biofouling. Faced with the increasing economic importance of these industries, it has become necessary to enhance membrane performance, so that these systems can become more affordable and efficient [6]. 

During filtration processes, fouling is the main problem causing loss of productivity, especially in the case of biological fluids. Indeed, it reduces equipment efficiency, increases production cost by repetitive cleaning and can induce contamination problems due to the growth of microorganisms at the membrane surface [7]. This has led to extensive research into the characterization of fouling. Most of the studies have focused on the evolution of permeate flux and total resistance *versus* time [7]. Recent researches focused on the role of several important factors affecting membrane fouling, such as cell characteristics (shape, size *etc*.), membrane properties, process parameters and hydrodynamic forces [8,9]. As yeast is one of the most important hosts of genetic modification for bioproduct manufacture and is also used in many industrial processes such as for beer, wine, bread *etc*. *Saccharomyces cerevisiae* microfiltration has appeared as a pertinent model for the study of fouling mechanisms. Indeed, recent scientific and industrial studies dealing with fouling mechanisms in the brewing and wine industry indicate the scientific and economic stakes of this application. In order to model real processes more closely, some studies were carried out with a *Saccharomyces cerevisiae* suspension combined to different proteins. Thus, Foley *et al.* [10] filtering yeast suspension of *Saccharomyces cerevisiae* (2 g L^−1^) on flat sheet membranes demonstrated that the deposition of particulate matter at the membrane surface could reduce access of fouling material inside the pores. The effect of interaction between yeasts or cell debris and molecular compounds such as proteins or lipids and their consequences on fouling have been pointed out in packed bed processes [4,11,12]. Bovine serum albumin (BSA) and yeast particles on the reversibility of cake formation during microfiltration were investigated [13,14]; these studies have suggested that protein fouling of the membrane and filtration resistance was reduced in the presence of cells. The same results were reported for other proteins in mixtures with *Saccharomyces cerevisiae* such as lysozyme and ovalbumin [15]. Yet, in the cases of dextran and yeast [16] or molasses and yeast [17], more compact cakes were formed during filtration of the mixture inducing the increase of resistance. Baker’s yeast was even combined with deproteinized cheese whey powder [18]. 

Concerning *Escherichia coli*, which is another widely cultivated microorganism and often considered as the prokaryotic model, the microfiltration of a fermentation broth containing *Escherichia coli*, studied by Li *et al.* [19] using a ceramic membrane filter with a nominal pore size of 0.2 µm indicated that the filtration resistance was mainly caused by the cake formed on the membrane surface. According to Okamoto *et al.* [20], the ultrafiltration resistance of *Escherichia coli* whole broth nearly doubled over 12 h of experiment. 

However, in many cases of application of microfiltration processes, heterogenic microbial and/or particle suspensions need to be considered; which is the case during the clarification of fermented beverages in which bacteria and yeast have to be removed [21], in membrane bioreactors for wastewater treatment or in biotechnology (production of pharmaceutical compounds by microorganisms). In most of these industrial applications, the main goals are not only to maintain a high permeate flow rate, but also to guarantee a constant quality of the product, *i.e.*, to control the retention of molecular compounds (for instance in the pharmaceutical industry). Thus the analysis of the mechanisms involved in filtration cake building and transport properties during the microfiltration of a defined mixture of model micro-organisms and/or particle suspensions could be of interest. Many studies have shown that cake resistance and porosity are often sensitive to cell and/or particle size, morphology, surface properties, ionic environment, medium components and interactions phenomena [9]. Indeed, McCarthy *et al.* studying the polymorphic yeast *K. marxianus* var. *marxianus* NRRLy2415, found a strong relationship between cell morphology (ovoid or filamentous) and the specific cake resistance [22]. Moreover, Mota *et al.* analyzed the specific cake resistance, porosity and tortuosity for rod-like particles (*B*. *subtilis*, *B*. *brevis* and *B*. *cereus*) spheroid cells (*Saccharomyces cerevisiae*) [1]. They showed that tortuosity due to cell shape was an important parameter for compressible cakes and that specific cake resistance of rod-like particles in cross-flow filtration depended on the higher tortuosity obtained by the shear-induced ordered arrangement. In turn, spheroid cells do not affect tortuosity as much as the rod-shaped cells. 

To our knowledge, only a few studies have investigated the microfiltration of a mixture of *Saccharomyces cerevisiae* and* Escherichia coli* suspensions at similar concentration ranges to those chosen in this work. These model suspensions were chosen as a first step of investigation in viewing the effect of microorganisms on filtration performance. Indeed, this paper focuses on the performance of microfiltration and the properties of the formed fouling by *Saccharomyces cerevisiae* and *Escherichia coli* on the membrane. Special attention was paid to the hydraulic resistance of the cake and its reversibility. The impact of a yeast/bacteria mixture was experienced and is discussed. 

At the microscopic scale several techniques have been already proposed to characterize cake properties [23]. One of them is Confocale Laser Scanning Microscopy (CLSM) which is becoming well established to analyze filter cakes. However the major of works are restricted to the examination *ex situ* of fouled filter samples after a sequence of filtration [24,25]. In the present paper, a new filtration chamber design which allows the direct observation of the formation of fouling *in situ* at a microscopic scale by CLSM microscopy has been developed and validated. 

## 2. Materials and Methods

### 2.1. Growth Media

#### 2.1.1. *Escherichia coli*

*Escherichia coli* strain (Top 10) used in this work, provided by the Center of Biotechnology of Sfax, was grown in Luria-Bertani (LB) medium. It consists of 10 g L^−1^ peptone, 5 g L^−1^ yeast extract and 5 g L^−1^ NaCl prepared in distilled H_2_O and adjusted to pH 7.2. For bacteria preculture fermentation, a few colonies were inoculated into 25 mL of the medium in a 50 mL flask and incubated at 37 °C under agitation (Ika Ks 130, Ika Werke GMBH & CO. KG) for 10 h. For the main culture fermentation, the preculture broth was inoculated into 500 mL of medium at a temperature of 37 °C under agitation over 7 h. At the end of this growth process, the suspension concentration was about 0.86 g L^−1^.

Cells from the fermentation broth were harvested by centrifugation at 6082 g for 15 min (Universal 320 R, Hettich Zentrifugen, Germany) then washed twice by suspension in water 9 g L^−1 ^NaCl followed by centrifugation. 

#### 2.1.2. *Saccharomyces cerevisiae*

Although it is now known that the results obtained with rehydrated yeast suspension could differ from those obtained with fresh cultivated cells, dehydrated yeast was used for the sake of simplicity. However a careful protocol was followed before use. The yeast suspensions were obtained from a dry strain of *Saccharomyces cerevisiae* (Alimentary Industry Rayen’s yeast, lot 2/153) rehydrated in water 9 g L^−1^ NaCl. The initial feed suspension was prepared as follows: the dry yeast was rehydrated at 35 °C for 10 min with stirring. In commercial baker’s yeast, some cell debris and soluble components are present. Their exact amount and composition are hard to analyze, which make it difficult to understand the influence of these soluble components in the yeast cake. Fortunately, broken cells and soluble components are lost in the supernatant during the washing [14]. After the rehydration, the yeast suspension was centrifuged for 15 min at 2376 g using a centrifuge (Universal 320 R, Hettich Zentrifugen, Germany) and washed twice with distilled water. Each wash step consisted of suspending the yeast in physiologic water followed by centrifugation under the same conditions as those mentioned previously.

For CLSM experiments, fresh cultivated yeast was used. The *S. cerevisiae* was grown in YPD medium at 30 °C under agitation (10 g L^−1^ glucose, 10 g L^−1 ^yeast extract, and 10 g L^−1^ bactopeptone) prepared in distilled H_2_O for 15 h. The final concentration was about 2.4 × 10^6^ cell L^−1^.

### 2.2. Dry Mass of Microbial Suspensions

During fermentation, dry bacterial mass was determined by centrifugation of the samples for 15 min at 2376 g (centrifuge Universal 320 R, Hettich Zentrifugen, Germany) and washing twice with distilled water. The mass was measured after drying the washed bacteria at 60 °C for 48 h. 

Dry yeast mass was measured after drying the washed yeast at 60 °C for 48 h. After repeating three times the same process for several yeast concentrations, the result was obtained as Equation (1):

Washed Yeast Dry mass (g L^−1^) = 0.732 × unwashed Dry yeast − 0.004 (with a R^2^ = 0.998) (1)

The yeast obtained after two washing steps was then suspended in physiologic water 9 g L^−1^ NaCl [14]. All yeast concentrations referred to in this paper are the concentrations of washed yeast in suspension.

The microbial concentrations of yeast and bacteria suspensions were also determined by absorbance at 620 nm wavelength (spectrophotometer HACH Lange DO, DR 5000). The correlations were obtained as Equations (2) and (3):

Bacteria dry mass (g L^−1^) = 0.599 × DO_620 nm_ − 0.006 (with a R^2^ = 0.995) (2)

Yeast dry mass (g L^−1^) = 0.925 − 0.234 × DO_620 nm_ (with a R^2^ = 0.995) (3)

### 2.3. Suspensions Preparation for Crossflow Microfiltration

As the filtration of cell suspension properties are sensitive to the cells’ age [20], all crossflow filtration experiments were conducted with fresh *Escherichia coli* culture. To ensure that crossflow filtration was done with microorganism at the same physiological state, trial fermentations were first performed. The dry mass of cell suspension at the end of the trial fermentation was determined. A new fermentation was then performed in identical conditions to produce the broth for crossflow filtration.

The suspensions of *Saccharomyces cerevisiae* were prepared just before the experiments. Table 1 summarizes the samples prepared for filtration runs and the abbreviations used for each of them.

**Table 1:** Summary of the suspensions prepared for microfiltration runs.

Abbreviation	*Saccharomyces cerevisiae* (g L^−1^)	*Escherichia coli* (g L^−1^)
S_1_	6	0
S_2_	6	0.15
S_3_	6	0.3
S_4_	8	0
S_5_	10	0
S_6_	10	0.15
S_7_	10	0.30

### 2.4. Fundamentals of Microfiltration

Darcy’s law [Equation (4)] models the solvent flow through a porous media during a filtration operation. Thus, the filtrate flux through the membrane was calculated by the following equations:

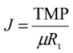
(4)

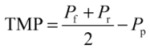
(5)
where *J* is the flux, TMP the transmembrane pressure, *μ* the dynamic viscosity of the feed, *R*_t_ the total resistance to flow and *P*_f_, *P*_r_ and *P*_p_ are respectively the pressure of the feed, the retentate and the filtrate. It can be observed that Equation (5) does not mean that TMP decreases linearly all along the membrane. Indeed, for a tubular channel membrane, complex spatial variations of pressure can take place both in the inner and outer region of the capillary. See for instance the effect of packing density in hollow fiber modules [26]. Nevertheless, it is a reasonable definition for TMP as *P*_f_, *P*_r_ and *P*_p_ are the only measurable parameters in the experiments.

The total resistance of the membrane (*R*_t_) can be considered as the sum of several resistances given by:

*R*_t_ = *R*_m_ + *R*_f_ = *R*_m_ + *R*_rf_ + *R*_if_ (6)

where *R*_m_ is the intrinsic resistance of clean membrane, *R*_f_ is the fouling layer resistance. 

Using Equation (6) assumes that there is separation of the scales, *i.e.*, particle size is much larger than pore size and that fluid flow is locally uniform at the porous surface. These assumptions can fail in some intricable cases [27,28], exhibiting a total resistance of one order of magnitude higher than the one obtained by Equation (6). The fouling layer resistance is negligible for a clean membrane but will increase with accumulation of fouling. *R*_f _can be considered as the sum of the external and the internal fouling resistances. The *R*_rf_ resistance is due to concentration polarization if particles are Brownian and convective deposition of solids (cake layer) on the membrane surface, and therefore it can be removed by cleaning with water after the filtration run. On the contrary, the *R*_if_ resistance is due to pore blocking and adsorption of materials on the membrane surface and pores which cannot be removed by water cleaning [29]. The intrinsic and the fouling layer resistance for the membrane were calculated using the following equations:

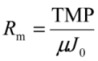
(7)

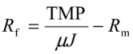
(8)
where *J*_0_ is the flux of distilled water at the beginning of the filtration experiment [30].

### 2.5. Crossflow Microfiltration Set-Up and Membrane

**Figure 1:**
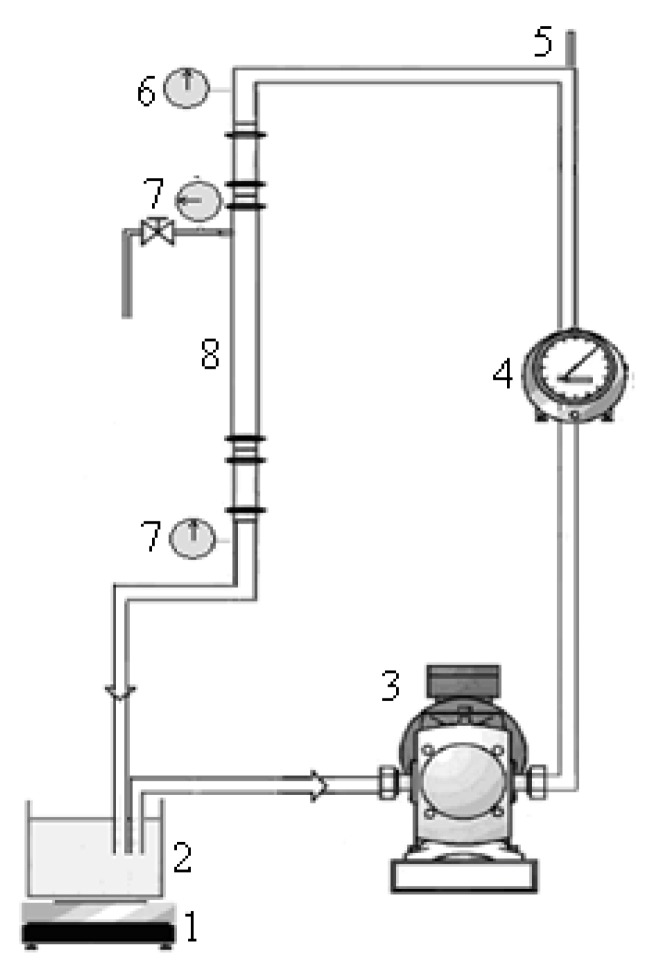
Scheme of crossflow filtration system, 1: Heating plate; 2: Feed tank; 3: Feed volumetric pump; 4: Flow meter; 5: Temperature gauge; 6 and 7: Pressure gauges; 8: Membrane module.

Microfiltration experiments were performed using a semi industrial device (Tech-Sep, Rhône Poulenc, France) equipped with a Kerasep membrane (illustrated in Figure 1) and fed from an 8 L feed tank with a volumetric pump (SKFD/005 Motovario, Spezzano, Italy). The ceramic membrane consists of 19 channels, each capillary having an internal diameter of 2.5 × 10^−3^ m and a length of 0.4 m; the area is 595 × 10^−4^ m^2^. The mean pore diameter is 0.2 µm. Before each experiment, the integrity of the membrane was checked by measurement of its permeability with distilled water at 20 °C. 

In agreement with a previous experiment performed with wastewater (data not shown), the filtration parameters selected for this work were: transmembrane pressure (TMP) of 1.5 bar and a cross flow velocity of 2.38 m s^−1^.The Reynolds number was calculated by Equation (9):

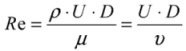
(9)
where *U* is the mean fluid velocity, *μ* is the dynamic viscosity of the fluid, *ν* is the kinematic viscosity (*υ* = *μ*/*ρ*), *ρ* is the density of the fluid and *D* is the hydraulic diameter of the channel. The flow is turbulent as the Reynolds number is equal to 5950. For the clean membrane, the flux obtained with distilled water was about 1200 L h^−1^ m^−2 ^at a TMP of 1.5 bar.

For this study, the experimental temperature was constant and equal to 25 °C. The crossflow velocity and the pressure were adjusted manually by the use of valves before and after the microfiltration carter. 

The volume reduction ratio (VRR) was calculated using the equation:

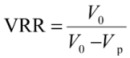
(10)
where *V*_0_ is the feed initial volume and *V*_p_ is the filtrate volume.

The flux loss was calculated using the equation:

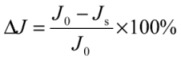
(11)
where *J*_0_ is the initial filtration flux and *J*_s_ is the stabilized filtration flux.

### 2.6. Membrane Regeneration

In order to recover the initial permeability, the MF membrane was carefully cleaned immediately after each experiment at a transmembrane pressure (TMP) of 1.5 bar and a crossflow velocity (*U*) of 2.38 m s^−1^. The pilot unit was rinsed with hot water (50 °C). After this first rinsing step, the water flow rate under the processing conditions (temperature, pressure) was measured to estimate the irreversible fouling (*R*_if_) of the membrane (due to a layer that could not be removed with water) using Equation (8). Then, a 10 g L^−1^ sodium hydroxide solution (80 °C during 30 min) was circulated. After a water rinsing step, a 5 mL L^−1^ nitric acid solution at 60 °C was circulated during 30 min. Finally, the circuit was rinsed with distilled water.

The total resistance (*R*_t_) was calculated with the stabilized filtrate flow rate using Equation (4). The resistance caused by concentration polarization and cake layer was estimated according to Equation (6). 

### 2.7. Scanning Electron Microscopy (SEM)

Specimens of microbial suspensions sampled at the end of the runs were observed with a scanning electron microscope Philips XL30 (Philips, Leimeil-Brevannes, France). The samples, deposited on small fragments of a Carbosep membrane (0.14 µm mean pore diameter, Tech-Sep, Rhône Poulenc, France), were fixed with glutaraldehyde and incubated overnight in the dark at 4 °C. The samples were then dehydrated with an ethanol/water gradient (25%–100%) and dried to CO_2 _critical point using a Baltec CPD 030 apparatus and coated with gold by a Baltec MED 20 apparatus (Balzers Union, Balzers, Germany).

### 2.8. Confocal Laser Scanning Microscopy Analysis

#### 2.8.1. Preparation of Microbial Suspensions

The microorganisms were double stained with Fluorescein diacetate (FDA) and Rhodamine 123 (Sigma Aldrich, St. Louis, MO, USA). The Fluorescein diacetate is a colorless compound which is hydrolyzed by both free and membrane bound enzymes releasing a colored end product. Rhodamine 123, is a cell permeant fluorescent dye that is sequestered by active mitochondria within a few minutes. Dye stock solutions were prepared as follows and stored at −20 °C: 0.2 mg mL^−1^ rhodamine 123 in water and 5 mg mL^−1^ FDA in acetone.

The *S. cerevisiae* was grown in YPD medium at 30 °C and the *E. coli* was grown on LB medium at 37 °C, as described previously. The two microorganisms were grown to the log phase (7.64 × 10^7^ cells mL^−1^ for yeast and 2 × 10^8^ cells mL^−1^ for bacteria). A sample of 20 mL of each broth was centrifuged at 10,621 g for 5 min at room temperature. Then, the supernatant was removed and the pellet was resuspended and washed with deionized water. Firstly, each pellet was stained with 1 µg mL^−1^ rhodamine, kept in the dark to avoid eventual bleaching for 5 min and centrifuged (10,621 g, 5 min). Then, the dyed cells were stained again with FDA (0.35 µg mL^−1^ PBS) following the same staining procedure. The supernatant was removed each time to remove excess dye and eliminate extracellular compounds.

#### 2.8.2. Microscopic Apparatus and Operating Conditions

Microscopic observations were performed on a Confocal Laser Scanning Microscope, CLSM, (LEICA SP2, DMRXA2) equipped with detectors and filter sets for the simultaneous monitoring of the different dyes used. The deposits were magnified by a ×40 objective. The samples were first analyzed in reflection mode to select the position of the *z*-axis corresponding to the membrane surface. Then, all the samples were analyzed using the fluorescence mode with the appropriate laser wavelength. In order to obtain a better-resolved image, noise was decreased by averaging many scans of each *z*-image. The result of CLSM analysis was *z*-series images which were used to carry out the *z*-projection and 3D orthogonal views. In the selected conditions, the voxel sizes were 0.73 µm × 0.73 µm × 0.48 µm with a zoom of 1 and 0.32 µm × 0.32 µm × 0.32 µm with a zoom of 1.44 during the observation of *E. coli* and the fouled microsieve with *S. cerevisiae*, respectively.

### 2.9. Image Analysis

The recorded pictures were processed using Image J (1.45 s downloaded from [31]). This program was used to measure the surface porosity. Indeed, the image was segmented by setting a threshold, which separated the pixels of interest from the rest of the image. Moreover, Image J enables a single 3D projection of the fluorescence-image series to be formed. The 3D image was projected to give a profile view. 

## 3. Results and Discussion

The sizes of the selected microorganisms were much larger than the membrane pore diameter; the mean diameter of the yeast was 5 µm, while the rod shape bacteria dimensions were 1 µm × 2.5 µm. Consequently, mainly external fouling could be expected.

### 3.1. Microfiltration of Cells Suspensions

The first runs were performed with yeast suspensions in order to investigate the influence of the variation of initial biomass concentration on membrane fouling nature. Further runs were investigated with yeast and bacteria mixture and were compared to first runs (Table 1). 

#### 3.1.1. Microfiltration of Yeast Suspensions

The filtrate fluxes of yeast suspensions filtered at different yeast initial concentrations (S_1_: 6 g L^−1^, S_4_: 8 g L^−1^, S_5_: 10 g L^−1^) were firstly investigated. 

**Figure 2:**
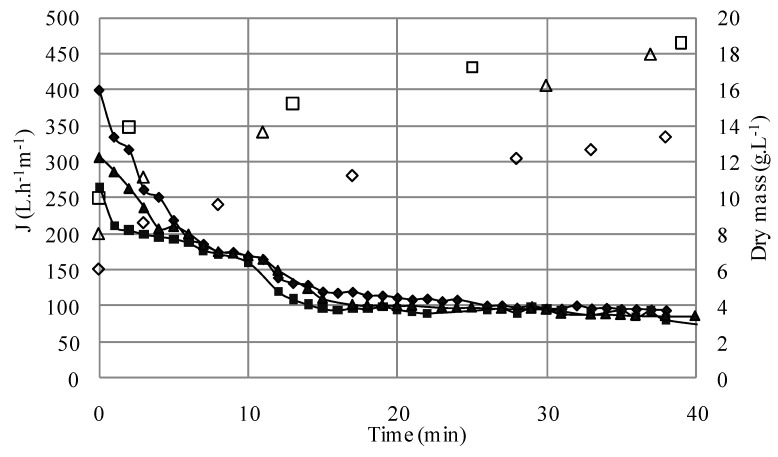
Effect of initial cell concentration (◆S_1_; ▲S_4_; ■S_5_) on microfiltration performance and evolution of dry mass of the retentates (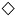
S_1_; ΔS_4_; □S_5_) *versus* time (TMP = 1.5 bar, *U* = 2.38 m/s, *R*e = 5950).

For the tested suspensions, high initial fluxes are observed followed by a rapid decline. The initial value of the permeate flux was inversely proportional to the cell concentration; for instance, an increase in cell concentration from 6 to 10 g L^−1^ allowed the decrease of initial filtrate flux from 400 to 265 L h^−1^ m^−2^ approximately. Then after approximately fifteen minutes of filtration, a pseudo-steady state was reached (Figure 2). The flux value at this steady state was quite similar, whatever the cell initial concentrations were the filtrate flux decreased, and was thus more pronounced with the increase of initial cell concentration. This result contradicts Russotti *et al*. [32] who concluded that, when initial cell concentration increases, cell mass deposited per filter surface area unit increases which leads to a lower steady state flux.

**Figure 3:**
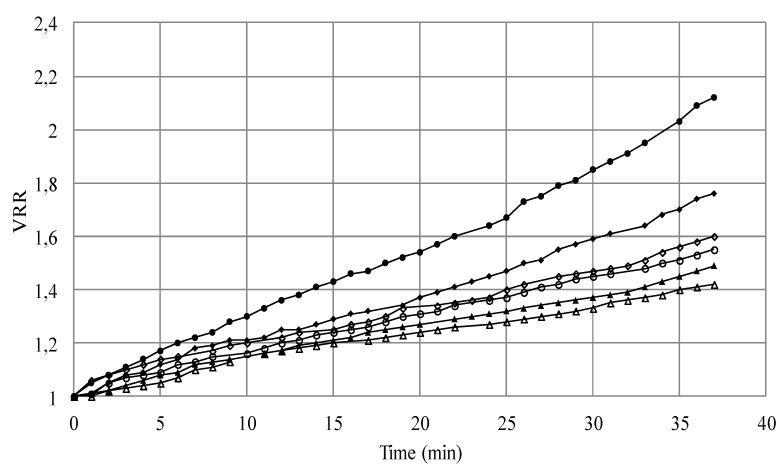
VRR evolution during the microfiltration of the microbial suspensions (●S_1_; ◆S_2; _◊S_3_; ○S_5_; ▲S_6_; ∆S_7_) (TMP = 1.5 bar, *U* = 2.38 m/s, *R*e = 5950).

During the filtration runs, the VRR increased from 1 to 2.12 and from 1 to 1.55 for S_1_ (the less concentrated suspension) and S_5_ (representing the most concentrated suspension), respectively (Figure 3). Furthermore, an increase of total solids in the retentate is noticed. This augmentation resulted in fouling and, consequently, contributed to the decline of the filtrate flux. 

**Figure 4:**
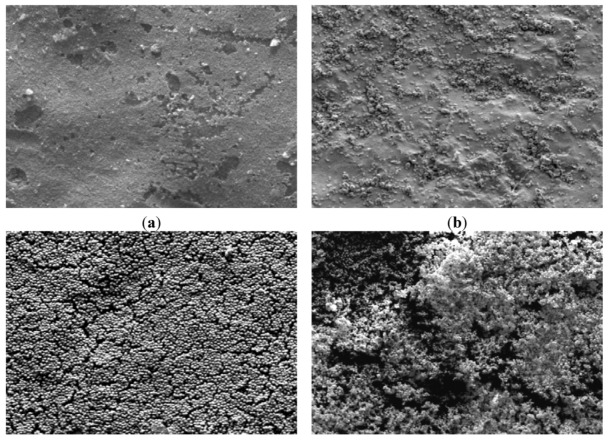
SEM images of clean membrane and retentates of suspensions containing different concentrations of *S. cerevisiae* (**a**) clean membrane; (**b**) S_1_; (**c**) S_4_; (**d**) S_5_ (×160).

Moreover, the increasing initial cell concentration could lead to an increase in cell aggregation in the retentate. Indeed, the retentate yeast organization that was visualized with SEM observations (Figure 4) for different concentrations could explain the increasing fouling behaviour of the suspensions. In these observations it can be seen that the size and shape of the suspended microorganisms are quite homogenous. 

The membrane fouling plays a key role in filtration processes. The presence of its effect was confirmed by the decline of the filtrate flux with processing time which lowered the filtration performances. This phenomenon could be due to several factors, such as cake formation, adsorptive fouling and pore blocking mechanisms [7] which may change the membrane characteristics and lead to an increase of filtration resistance. In order to understand the nature of fouling on the filtrate flux drop with time, total resistance, intrinsic membrane resistance and the nature of the fouling resistance were determined. It can be noted that, effectively, the contribution of intrinsic membrane resistance, *R*_m_ = 0.448 × 10^12^ m^−1^, was rapidly negligible compared to the fouling resistances as shown in Table 2.

**Table 2:** Values of resistances obtained from the microfiltration of tested cell suspensions.

Suspension	Resistances ×10^12^ (m^−1^)				Normalised resistances (%)	
	*R*_t_	*R*_f_	*R*_if_	*R*_rf_	*R*_if_/*R*_f_	*R*_rf_/*R*_f_
S_1_	5.79	5.34	4.01	1.33	75	25
S_2_	6.40	5.95	2.83	3.12	48	52
S_3_	6.49	6.04	2.39	3.65	40	60
S_4_	6.24	5.78	2.67	3.12	46	54
S_5_	6.63	6.18	1.65	4.53	27	73
S_6_	6.72	6.27	2.51	3.76	38	62
S_7_	7.68	7.23	2.84	4.39	40	60

**Figure 5:**
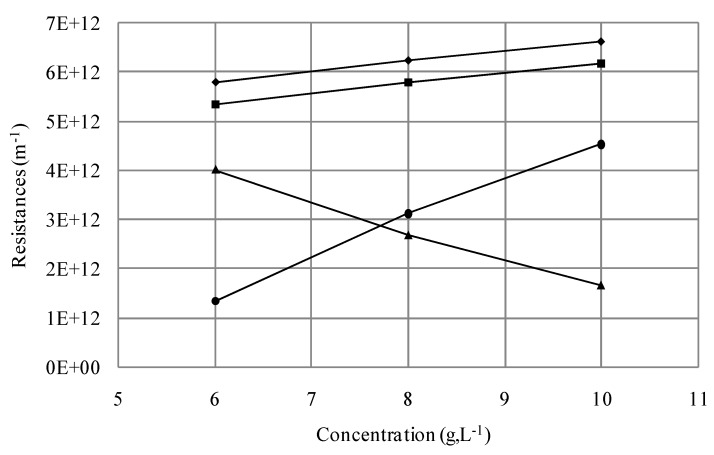
Effect of initial cell concentration of *S. cerevisiae* on cake resistances (◆*R*_t_; ■*R*_f_; ▲*R*_if_; ●*R*_rf_).

To assess the potential reversibility of fouling, the resistances for the different concentrations were compared (Figure 5). Indeed, fouling and reversible resistances increased linearly with higher concentration. However, R_if_ decreased linearly when the concentration of the suspension increased. 

As previously mentioned, aggregation phenomena at high yeast concentrations, suggested by SEM observations (Figure 4), could contribute to the membrane fouling.

#### 3.1.2. Microfiltration of Yeast and Bacteria Mixture

With the addition of *Escherichia coli* to the feed suspension, the interactions between yeasts cells and bacteria on the nature of fouling could be investigated. In this study, we focused on the suspensions presenting the highest reversible fouling (S_5_) and the suspension causing the highest irreversible one (S_1_).

##### 3.1.2.1. Effect of Initial Cell Concentration on Microfiltration Performances

**Figure 6:**
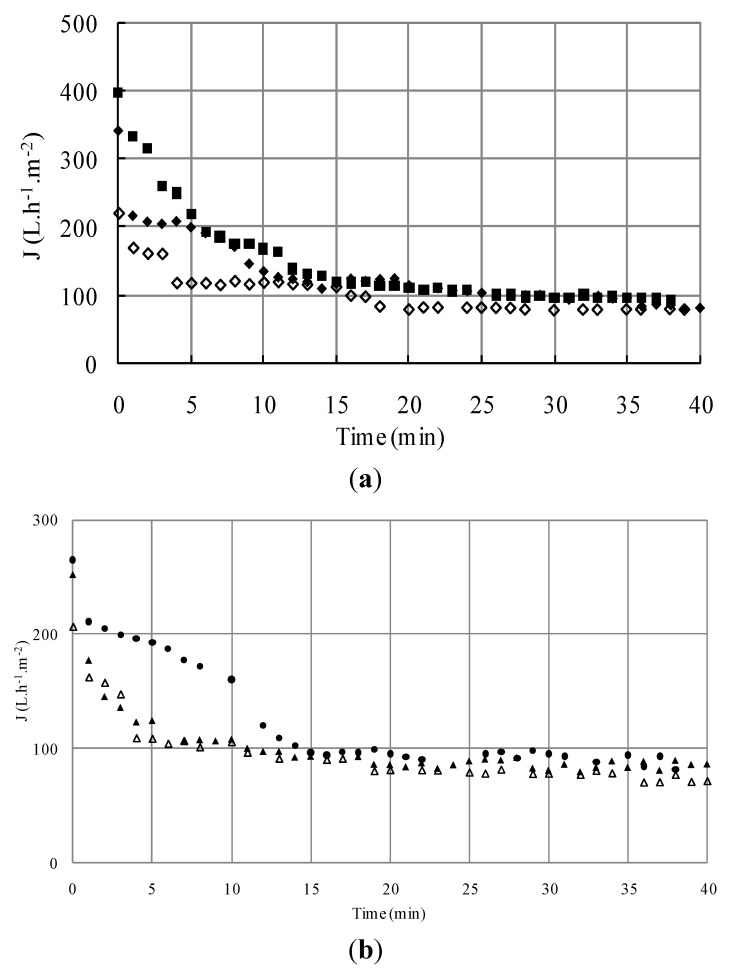
(**a**) Effect of initial cell concentration on microfiltration of the mixture: *S. cerevisiae* (6 g L^−1^) and *E. coli* [0 g L^−1^ (■S_1_); 0.15 g L^−1^ (◆S_2_); 0.3 g L^−1^ (◊S_3_)] (TMP = 1.5 bar, *U* = 2.38 m/s, *R*e = 5950); (**b**) Effect of initial cell concentration on microfiltration of the mixture: *S. cerevisiae* (10 g L^−1^) and *E. coli* [0 g L^−1^ (●S_5_); 0.15 g L^−1^ (▲S_6_); 0.3 g L^−1^ (ΔS_7_)] (TMP = 1.5 bar, *U* = 2.38 m/s, *R*e = 5950).

The effects of small bacteria on the type of cake formation could be studied by the addition of *Escherichia coli* to the microbial mixture. Figure 6a,b shows the flux evolution of two concentrations of washed yeast (6 g L^−1^and 10 g L^−1^) mixed with two different bacterial suspensions (0.15 g L^−1^and 0.30 g L^−1^). At the lower yeast concentration of 6 g L^−1^ (Figure 6a), the addition into the feed suspension of 0.15 g L^−1 ^*Escherichia coli* reduced the initial flux value but after five minutes of filtration, the obtained flux was similar despite bacteria addition. The same behavior could be observed for the addition of 0.3 g L^−1^ of *Escherichia coli* with a more pronounced decrease in initial flux value and a slightly smaller value in the stabilized flux. In all runs for single species suspensions, the flux decreased until the fifteen first minutes was reached. 

Under the same operating conditions, fluxes of yeast and bacteria mixtures were significantly lower in the case of the higher yeast concentration of 10 g L^−1^ (Figure 6b). The fluxes for mixture suspensions show an important decrease until the fifth minute and, then, tend towards stability. The percentages of flux loss during the different runs S1, S2, S3, S4, S5, S6 and S7 were respectively 77% ± 0.8%, 77% ± 1.5%, 65% ± 0.8%, 72% ± 0.6%, 69% ± 1.1%, 66% ± 1.1% and 66% ± 0.5%. That showed that the flux loss was higher than 65% in the case of all cell suspensions. 

The size of *Escherichia coli* cells is about 0.5–1 µm in diameter and 2.5 µm in length [19] while the yeasts are ellipsoidal cells with a mean diameter around 5–6 µm. Due to their low size, bacteria cells can be potentially captured on the bottom or inside of the yeast cake, which can increase the fouling resistance [23]. Indeed, the study of microfiltration on a ceramic membrane filter of *Escherichia coli* fermentation broth by Li *et al.* [19] showed that the flow resistance was mainly caused by the cake formed on the membrane surface. This author concluded that the contributions due to internal pore blockage by cells and the membrane itself are relatively unimportant. Indeed, the high ratio bacteria size/membrane pore diameter could explain the low internal fouling. It should also be mentioned that interactions between yeast/bacteria or bacteria/membrane could participate in the consolidation of the cake structure. 

##### 3.1.2.2. Effect of Initial Cell Concentration on Resistances

Interactions between microorganisms themselves and microorganisms/membrane could increase cake resistance by modifying the cake pore structure and by changing the cells aggregation [20,33,34]. As expected, with the introduction of *Escherichia coli*, the fouling resistance increased, compared to the one obtained with pure yeast suspensions. The presence of the bacteria modified the reversible part of the fouling even though it did not affect the flux. Indeed, the addition of bacteria to the suspensions of 6 g L^−1^ of yeast caused increase of reversible fouling: the ratio *R*_rf_/*R*_f_ increased from 25% for S_1_ to reach 52% and 60% for S_2_ and S_3_, respectively (Table 2). Moreover, in the case of high yeast concentration (10 g L^−1^), even though the reversible fouling remained the most important which decreased with the rise of the bacteria concentration: the ratio *R*_rf_/*R*_f_ decreased from 73% for S_5_ to reach 62% and 60% for S_6_ and S_7_, respectively. The tendency was inversed for the irreversible fouling (Table 2). Even although some studies have concluded that internal fouling was negligible, others have shown that bacteria deformation could led to pore blocking, depending on membrane properties [35,36]. Indeed, when approaching the entrance of a pore, bacteria are submitted to hydrodynamic stresses due to velocity and pressure fields in the fluid that surrounds them. Such stresses could lead to their volume reduction (related to the osmotic equilibrium) and surface deformation (governed by the cell-wall Young modulus value) which would allow the cell to penetrate into the membrane pore (pore size deformation around mean diameter). In a wider sense, many authors assume bacteria deformation at constant volume. Indeed, some reports indicate that a low ionic strength or a high negative charge of bacteria facilitate their transport through a porous medium [37,38]. Moreover, bacteria volume reduction was observed in other studies. Mille *et al.* showed that, when submitted to a mechanical pressure, bacteria could lose a part of their internal liquid [39]. Others focused on the ability of various bacteria to pass through a membrane with a nominal pore size smaller than the cell size [36,40]. While Suchecka *et al.* [40] assumed that the passage of a Gram negative bacteria through a microfiltration membrane is possible by releasing intracellular matter into the environment. Lebleu *et al*. [36] found a correlation between the bacteria external structure and the filtration behavior. Indeed, for many tested strains, only the Gram-negative ones were able to pass through the 0.4 µm membrane whereas all Gram-positive ones were rejected.

**Figure 7:**
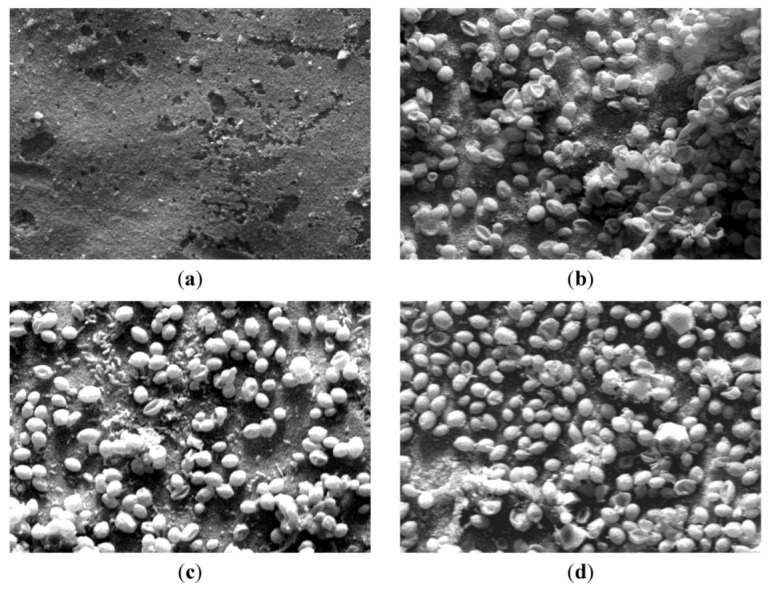
SEM images of the clean membrane and retentates of solutions containing different concentrations of *S. cerevisiae* and *E. coli* (**a**) clean membrane; (**b**) S_1_; (**c**) S_3_; (**d**) S_7_; (×1000).

At this point, the observation by SEM of the characteristics of the retentates sampled during filtration runs is highly interesting as it allows the retentates characteristics to be observed (Figure 7). Comparativey to the clean membrane piece used as a support (Figure 7a), the suspension’s retentate containing only *Saccharomyces cerevisiae* S_1_ (Figure 7b) at the lower tested concentration showed mostly individualized yeast cells; only a few aggregates and dead cells can be seen. When 0.3 g L^−1^* Escherichia coli* were added (S_3_), individualized yeasts with a few aggregates (Figure 7c) with yeast and bacteria apart were observed. Moreover, S_7_ observation (Figure 7d) illustrated a higher cell density caused by higher concentration of *Saccharomyces cerevisiae* and more aggregates formed as well by *Saccharomyces cerevisiae* and *Escherichia coli.* These observations permit the assumption that *Escherichia coli* probably tend to favour aggregates and thus cake formation in comparison with pure suspensions of *Saccharomyces cerevisiae*.

To improve the analysis of the deposit characteristics during the microfiltration process, a proposed strategy was based upon direct observation with Confocal Laser Scanning Microscopy (CLSM). Indeed, different works have previously demonstrated the interest in CLSM to investigate fouling mechanisms at the local scale [12,23,41], performed thanks to a specifically designed microfiltration chamber equipped with a microscope glass coverslip. This system could then allow *in vivo* and *in situ* local analysis of the 3D-organisation of fouling deposition. 

### 3.2. Filtration Chamber Design

Even though the mechanisms leading to membrane fouling during filtration are not yet fully understood, macroscopic laws have been developed assuming that basic mechanisms take place such as pore clogging, cake formation *etc*. These models are widely used in order to explain flux decline. However, they do not take into consideration particle size distribution, pore size distribution, cake morphology *etc*. In order to improve the performance of membrane processes, these mechanisms have to be better understood at the microscale level. Recent studies [1,42,43] have pointed out the strong influence of particle and flow characteristics on the properties of the cake and the filtration performances. Moreover, in the case of suspended microorganisms with a size distribution, their location and arrangement inside the cake seems to be correlated to their size [23]. However, the used chamber design allowed only the observation of the upper part of the deposits, corresponding to a 30µm thickness slice. Moreover, the cake was obtained after the one-step filtration of a selected volume of microbial suspension and it was then subsequently observed. Besides, the progressive formation of the deposit (real time analysis of the monolayer cake) and the effect of hydrodynamics on deposit formation and organization were not realized. All these drawbacks should be solved in an upcoming study.

To better study the respective effects of flow conditions and particle size, shape and surface properties on the dynamics of cake growth and morphology at the particle level and to establish correlations with microfiltration performance, a specific filtration chamber design has to be developed and optimized. This design should allow the direct observation of the formation of the cake *in situ* under various flow conditions (dead-end or cross-flow filtration).

#### 3.2.1. Design Guidelines

Many studies have been conducted in the areas of membrane manufacturing and applications development. However, filtration chamber design is generally made by membrane system specialized companies and fewer investigations have been carried out on module development [23,33,44]. Our aim was to design a filtration chamber enabling a direct visualization of the deposit growth during filtration operating under controlled flow conditions. Indeed, the observation chamber previously used by Beaufort *et al*. [23] showed only a few µm of the top of the cake. In order to accurately measure the cake characteristics (thickness, porosity *etc*.), the chamber design should both allow the observation of the clean membrane and the cake growth during all the runs. Towards this end, the design should take into account several requirements and guidelines about chamber sizing, accurate resolution for observations and cleaning of the system.

**Figure 8:**
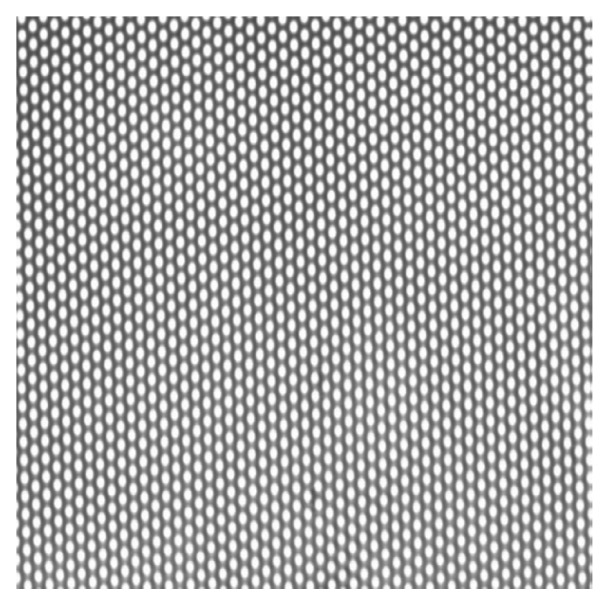
Optical micrograph of microsieve with circular pores: 0.45 µm.

1. The key-question was to find the optimal apparatus size which allows the observation *in situ* of all the deposit cake in its full depth and not only in a part of it. Moreover, the dimensions of the observation window should be sufficiently large to allow the microscope objective to scan a representative area of the filtration membrane. In addition, the chamber glass coverslip thickness has to remain as thin as possible to keep a total chamber depth suitable for the working length of the microscope objective. However, it should not be deformed when submitted to pressure variations due to fluid flow that limits the size of the window.

2. In order to observe the particle deposit on the membrane surface *in situ*, the light has to cross the glass cover slip and the particle suspension above the microsieve. The total distance corresponds to the height of the flow channel required to set the selected flow conditions over the membrane and the thickness of the cover slip. According to these constraints, a microscope objective with “Long Working Distance Lenses” has to be selected.

3. Membrane cleaning: The filtration chamber material has to be consistent with the membrane cleaning technique which most commonly requires two phases: hydraulic cleaning and chemical cleaning. 

4. Membrane choice: It should be noted that the microsieve system is the best compromise in terms of membrane area minimization and permeate flow rate maximization [45]. Indeed, microsieves are perfectly plane thin silicon nitride membranes with a regular arrangement of circular well-defined and uniform pores of high porosity and permeability, and compared to conventional membranes, exhibit a uniform distribution of particles in the filtration field. Figure 8 shows micrographs of the sieves with 0.45 µm pore width (Aquamarijn^®^, [43]). Moreover, these membranes have previously been used for yeast separation in beer processing [46].

In this research, a designed laboratory-scale filtration chamber incorporates the developments discussed above.

#### 3.2.2. Direct Observation Chamber and Apparatus

**Figure 9:**
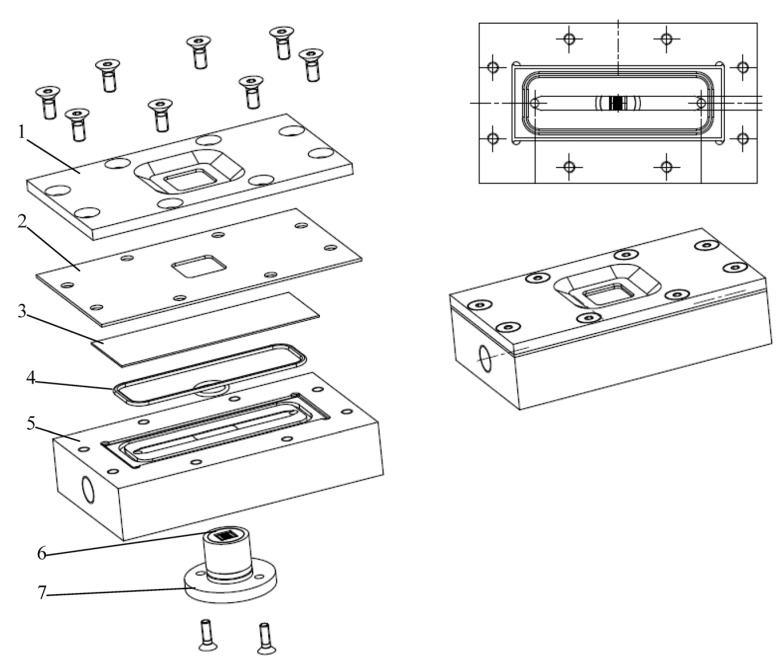
Schematic diagrams of the direct observation filtration chamber (1: cover; 2: joint; 3: glass coverslip; 4: O ring; 5: base; 6: microsieve; 7: disc mount).

**Figure 10:**
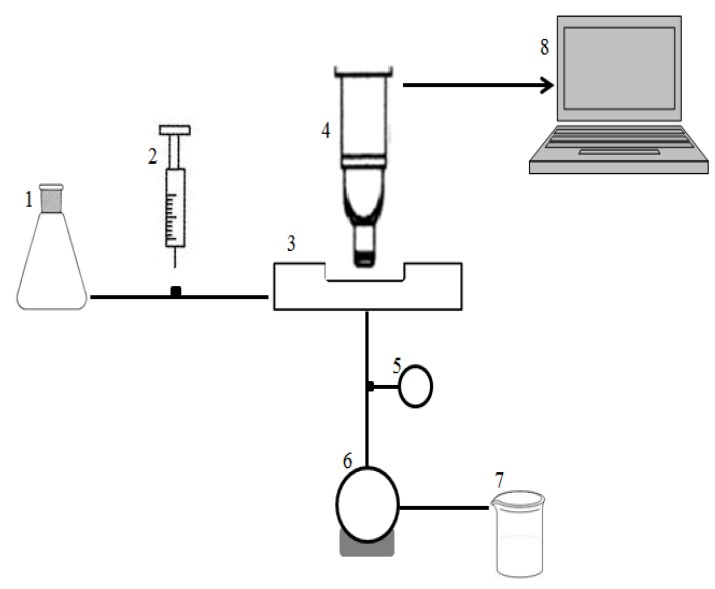
Schematic illustration of the microfiltration set up (1: deionized feed flask; 2: particle injection; 3: filtration chamber; 4: CLSM microscope; 5: numeric manometer; 6: pump; 7: permeate outlet; 8: PC).

The filtration chamber was designed and constructed from stainless steel with a glass window in the top plate allowing direct microscopic observation of particle deposition and cake formation. The selected material was stainless steel suitable for chemical cleaning. A schematic illustration of the chamber and the filtration unit are presented in Figure 9, Figure 10. For deposit observations during the filtration process, this chamber can be directly mounted on a CLSM microscope (Leica, Japan). Images of the membrane surface are taken and combined into an overlay with the Leica CLSM software. 

The filtration apparatus (Figure 10) consisted of two circulation loops with two feed flasks: The first one is for the circulation of deionized water and the second for the particle suspension. Moreover, a pump (Masterflex, Bioblock scientific, USA) and a digital compact vacuum meter (Thyrcont, Germany) are used to monitor the crossflow conditions.

#### 3.2.3. CLSM for Membrane Characterization and Preliminary Fouling Observations

The manufacturer’s data for surface porosity of the clean membrane was compared with the porosity determined experimentally in reflection mode, to check the measurement accuracy obtained by image processing of CLSM recordings. The values were 25.6% and 25.5% ± 2.3% respectively, which validate the use of CLSM and Image J image processing in order to get a correct estimation of cake properties.

The proposed strategy uses the direct observation by CLSM of fluorescent dyed microorganisms (yeasts and/or bacteria). The studied microorganisms were double stained. As the selected fluorescent dyes targeted different cell sites, the microorganism were efficiently stained. Fluorescence intensity is dependent on several factors including dye concentration, spectral intensity at a particular wavelength, photobleaching *etc*. Characteristics of the two dyes were examined and visualization conditions optimized. Furthermore, even though background fluorescence caused by the microsieve was frequently less intense than the dyed cells, visualization conditions were set to only observe the microorganisms.

**Figure 11:**
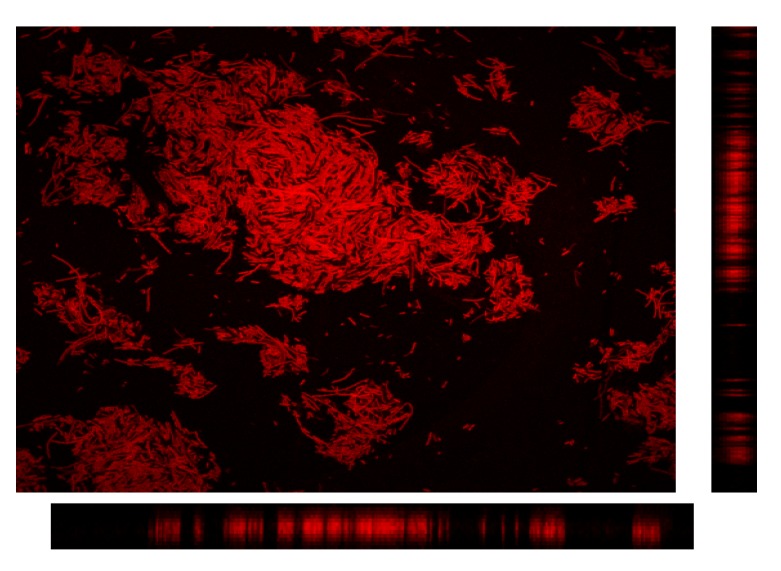
Orthogonal view of the 3D reconstruction of an *E. coli* fouling cake dyed by rhodamine 123.

**Figure 12:**
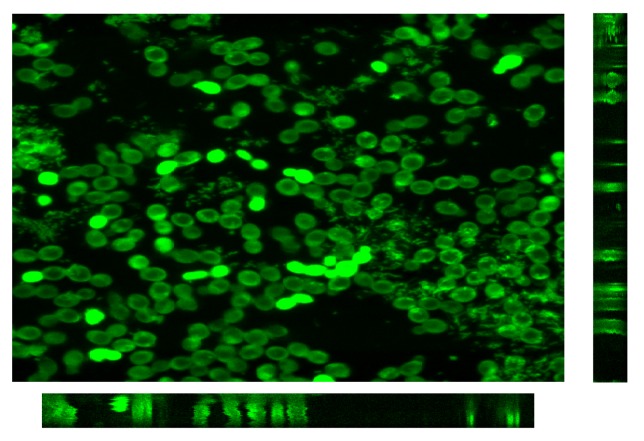
Orthogonal view of the 3D reconstruction of a 0.45 µm microsieve fouled by a *S. cerevisiae* suspension dyed by rhodamine 123 and fluorescein diacetate.

In order to validate the staining method, preliminary observations of different samples with CLSM were done. Firstly *Escherichia coli* aggregates were dyed with rhodamine 123 and directly observed on a glass slide. Secondly, a microsieve fouled by 4.5 × 10^−3^ g of *Saccharomyces cerevisiae* both dyed by rhodamine 123 and fluorescein diacetate, was examined. The stack of images recorded along the *z*-axis was processed with the software Image J to generate 3D images and, then, the aggregate or the deposit could be analyzed according to various cross sections. These orthogonal planes allow examination of the cross-sectional slices through the image volume in the *XZ*, *YZ* and *XY* planes (Figure 11, Figure 12, respectively). 

**Figure 13:**
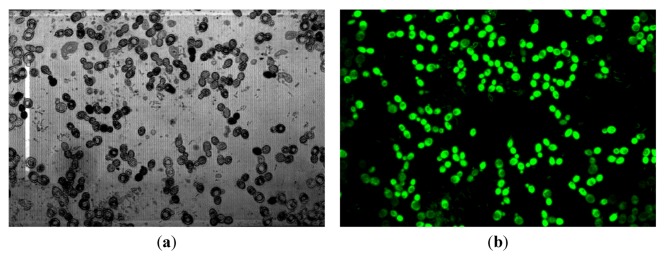
CLSM image in reflection and (**a**) fluorescence (**b**) mode in of a 0.45 µm microsieve fouled by *S. cerevisiae* suspension dyed by rhodamine 123 and fluorescein diacetate.

**Figure 14:**
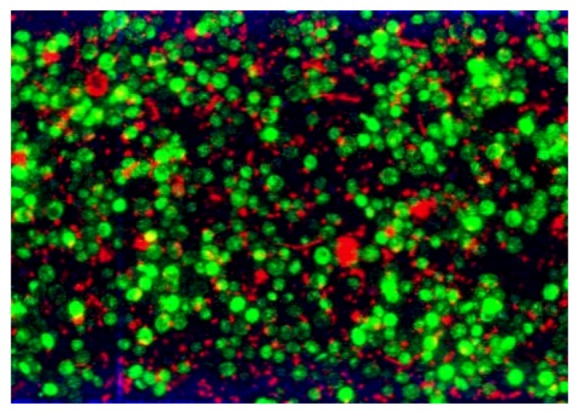
Composite image of a 0.8 µm pore size microsieve fouled with one layer of *E. coli* and *S. cerevisiae*.

Figure 13 shows the fouled microsieve by yeast in reflection mode which validated that CLSM makes it possible to clearly distinguish both the pores on the membrane surface and the cells deposited. In addition first tests of the filtration of a mixture of *Saccharomyces cerevisiae* and *Escherichia coli* were performed. A promising typical image of the first layer of the cake obtained by CLSM is shown in Figure 14. It is thus expected to be able to better understand the link between bioparticle properties and interactions, cake formation and membrane fouling and subsequent filtration performance.

These first results have shown that this new filtration chamber could be a powerful tool for fouling characterization and analysis of cake building mechanisms. 

## 4. Conclusions and Perspectives

The fouling behavior of the microorganisms was analyzed thanks to model suspensions. It was shown that membrane fouling is highly dependent on the respective concentration of microorganism species in suspension. Indeed, the microfiltration of *Saccharomyces cerevisiae* yeast mainly causes a reversible fouling. The addition of *Escherichia coli* to the lowest yeast concentration of 6 g L^−1^ induces an increase of reversible fouling. Moreover, the addition of *Escherichia coli* reveals a significant increase of irreversible resistance for higher concentration of yeast (10 g L^−1^). This analysis made at a macroscopic scale is in agreement with previous work of Beaufort *et al*. [23] obtained at a microscopic scale but limited to the upper part of a thick cake already formed (30 μm *vs.* 80 μm). To better understand the effects of incorporating bacteria into cake on the fouling type, the progressive formation of the deposit *in situ* should be studied in further work.

In order to improve the knowledge on the building mechanisms and morphology of microbial cakes, a new filtration chamber design allowing the direct *in situ* observation of the formation and subsequent organization of microbial deposits has been proposed. Moreover this device has been designed to operate either in dead-end or cross-flow filtration. This original approach for fouling characterization using CLSM and image analysis technique has been developed and validated. 

This device will give us the opportunity to exhibit the 3D-organisation of the microorganisms in the cake and then improve the study of fouling mechanisms in the case of bi-dispersed suspensions by the direct visualization of the fluorescent fouling *in situ* and the identification of each microorganism. 

The ongoing work intends to analyze the characteristics of mixed microbial deposits at the particle level under controlled flow conditions (transmembrane pressure and cross flow rate) in order to better understand fouling mechanisms and to improve the performances at the process level. Moreover, this first approach, performed with re-hydrated and rinsed microorganisms, should be developed for cultivated microorganisms since it is obvious that all the chemical compounds of the medium surrounding the cells could play an important part in cake organization and reversibility.

## Nomenclature

MF: microfiltration
UF: ultrafiltration
LB: Luria Bertani medium
YPD: Yeast Peptone Dextrose
VRR: volume reduction ratio
SEM: scanning electron microscopy
*R*e: Reynolds number
*D*: hydraulic diameter of the channel (m)
*J*: flux (L h^−1^ m^−2^)
*J*_0_: flux of distilled water for the clean membrane (L h^−1^ m^−2^)
TMP: transmembrane pressure (bar)
*R*_t_: the total resistance to flow (m^−1^)
*P*_f_: pressure of the feed (bar)
*P*_r_: pressure of the retentate (bar)
*P*_p_: pressure of the filtrate (bar)
*R*_m_: membrane intrinsic resistance of the clean membrane (m^−1^)
*R*_f_: fouling resistance (m^−1^)
*R*_rf_: reversible fouling resistance (m^−1^)
*R*_if_: irreversible fouling resistance (m^−1^)
*U*: tangential velocity (m s^−1^)
*V*_0_: feed initial volume (m^3^)
*V*_p_: filtrate volume (m^3^)
VRR: volume reduction ratio
*μ*: dynamic viscosity (Pa s)
*ρ*: fluid density (kg m^−3^)
*υ*: *υ* = *μ*/*ρ* kinematic viscosity
